# Phylogenomic Analysis of *Oenococcus oeni* Reveals Specific Domestication of Strains to Cider and Wines

**DOI:** 10.1093/gbe/evv084

**Published:** 2015-05-14

**Authors:** Hugo Campbell-Sills, Mariette El Khoury, Marion Favier, Andrea Romano, Franco Biasioli, Giuseppe Spano, David J. Sherman, Olivier Bouchez, Emmanuel Coton, Monika Coton, Sanae Okada, Naoto Tanaka, Marguerite Dols-Lafargue, Patrick M. Lucas

**Affiliations:** ^1^Univ. Bordeaux, ISVV, EA 4577 Œnologie, Villenave d’Ornon, France; ^2^Research and Innovation Centre, Fondazione Edmund Mach, San Michele all’Adige, Italy; ^3^BioLaffort, Research Subsidiary of the Laffort group, Bordeaux, France; ^4^Department of Agriculture, Food and Environment Sciences, University of Foggia, Foggia, Italy; ^5^INRIA, Univ. Bordeaux, Project team MAGNOME, Talence, France; ^6^CNRS, Univ. Bordeaux, UMR 5800 LaBRI, Talence, France; ^7^INRA, UMR444, laboratoire de Génétique Cellulaire, Castanet-Tolosan, France; ^8^GeT-PlaGe, Genotoul, INRA Auzeville, Castanet-Tolosan, France; ^9^Université de Brest, EA 3882, Laboratoire Universitaire de Biodiversité et Ecologie Microbienne, ESIAB, Technopôle Brest-Iroise, Plouzané, France; ^10^NODAI Culture Collection Center, Tokyo University of Agriculture, Japan; ^11^Bordeaux INP, ISVV, EA 4577 Œnologie, Villenave d'ornon, France

**Keywords:** *Oenococcus oeni*, genomics, phylogeny, population structure, domestication

## Abstract

*Oenococcus oeni* is a lactic acid bacteria species encountered particularly in wine, where it achieves the malolactic fermentation. Molecular typing methods have previously revealed that the species is made of several genetic groups of strains, some being specific to certain types of wines, ciders or regions. Here, we describe 36 recently released *O. oeni* genomes and the phylogenomic analysis of these 36 plus 14 previously reported genomes. We also report three genome sequences of the sister species *Oenococcus kitaharae* that were used for phylogenomic reconstructions. Phylogenomic and population structure analyses performed revealed that the 50 *O. oeni* genomes delineate two major groups of 12 and 37 strains, respectively, named A and B, plus a putative group C, consisting of a single strain. A study on the orthologs and single nucleotide polymorphism contents of the genetic groups revealed that the domestication of some strains to products such as cider, wine, or champagne, is reflected at the genetic level. While group A strains proved to be predominant in wine and to form subgroups adapted to specific types of wine such as champagne, group B strains were found in wine and cider. The strain from putative group C was isolated from cider and genetically closer to group B strains. The results suggest that ancestral *O. oeni* strains were adapted to low-ethanol containing environments such as overripe fruits, and that they were domesticated to cider and wine, with group A strains being naturally selected in a process of further domestication to specific wines such as champagne.

## Introduction

The lactic acid bacteria species *Oenococcus oeni* is present on grapes and other fruits at very low and often undetectable levels (Lonvaud-Funel 1999; Bae et al. 2006; Barata et al. 2012). It proliferates in wine and cider during or after the yeast-driven alcoholic fermentation and reaches population levels above 10^6^ cells/ml, thus becoming the only detectable bacterial species (Fleet et al. 1984; Lonvaud-Funel 1999). Its development in wine is desirable because *O. oeni* performs the malolactic fermentation (MLF), which mainly consists in the conversion of malate into lactate and carbon dioxide and improves the taste and overall quality of wine (Davis et al. 1985; Bartowsky 2005). *Oenococcus oeni* is often used as a starter culture in wine to better control the onset and duration of MLF. Starter strains are selected on the basis of their capacity to promote the transformation of malate in a panel of wines. This relies upon the tolerance of bacteria to stresses encountered in wine, such as acidity (pH 2.9–4.0), ethanol (10–15%), sulfites, or phenolic compounds (Torriani et al. 2011). The *Oenococcus* genus comprises two other species: *Oenococcus kitaharae*, found in composting distilled shochu residues (Endo and Okada 2006) and *Oenococcus alcoholitolerans*, recently documented from cachaça and bioethanol fermentation processes (Badotti et al. 2014). Although being adapted to alcohol-rich environments these species were not reported in wine and differ from *O. oeni* in that *O. kitaharae* lacks the ability to perform MLF (Marcobal et al. 2008) and *O. alcoholitolerans* produces acid from sucrose, a characteristic that is rarely found among *O. oeni* strains (Badotti et al. 2014; Dimopoulou et al. 2014). The first complete *O. oeni* genome sequence of strain PSU-1 revealed a reduced genome of 1,780,517 bp and a number of metabolic pathways involved in growth in wine, MLF, and aroma production (Mills et al. 2005; Makarova et al. 2006; Makarova and Koonin 2007). The sequences and comparative analysis of 13 additional genomes have extended the repertoire of industrially relevant genes contributing to wine tolerance and MLF (Borneman et al. 2010, 2012a). Interestingly *O. oeni* lacks the mismatch repair genes *mutS* and *mutL*. This atypical situation was also detected in the sister species *O. kitaharae* and correlated to the hypermutable status of both species (Marcobal et al. 2008). A BLAST search for *mutS* and *mutL* on *O. alcoholitolerans* does not show any significant match (data not shown). A mutation in *mutL* has also been reported in a fast evolving strain of *Lactococcus lactis* (Bachmann et al. 2012) It is anticipated that hypermutability is responsible for the high allelic diversity of *O. oeni* and contributes to the adaptation of the species to the wine environment. The population structure of the species was examined by multilocus sequence typing (MLST) of large collections of strains isolated from various products and places (Bilhère et al. 2009; Bridier et al. 2010). The strains form two genetic groups, namely A and B, possibly subdivided into subgroups linked to specific regions, such as Chile and South Africa, or products such as cider and champagne.

We have recently sequenced 36 additional genomes of strains isolated from diverse origins with the aim to compare their genetic equipment, particularly genes involved in exopolysaccharides production (Dimopoulou et al. 2014). In this study, we report the general features of these genomes and a phylogenomic analysis of all 50 *O. oeni* genomes reported to date. We also report three new genomes of *O. kitaharae* strains.

## Materials and Methods

### Bacterial Strains, Genomic DNA Isolation, and Polymerase Chain Reaction Conditions

All the strains analyzed in this study are listed in table 1 and available from the indicated culture collections. Two couples of polymerase chain reaction (PCR) primers specific for group A and B strains targeting genes of a cell surface protein precursor and a hypothetical protein, respectively, were designed using Primer3 (Koressaar and Remm 2007; Untergasser et al. 2012), evaluated with MFEprimer (Qu et al. 2009) and validated in the laboratory against a collection of 41 previously genotyped strains. For total DNA PCR, 65 wine samples were collected from 58 wineries of the Aquitaine region. DNA was extracted from a centrifuged pellet by mechanic lysis using glass beads, followed by Nuclei Lysis Solution and Protein Lysis Solution (Promega) and 10% PVP solution to eliminate phenols. Microbial DNA used for genome sequencing and colony PCR were extracted using the wizard genomic DNA purification kit according to manufacturer’s recommendation (Promega). PCR amplifications were performed in a reaction volume of 20 µl containing *Taq* Master Mix (BioLabs), a final concentration of 0.25 µM of primers and 2.5 ng of DNA. Sequences were amplified for 30 cycles.

**Table 1:** General Features of *O. oeni* and *O. kitaharae* Genomes

Strain^a^	Origin	Sequence data								Accession	References
		Method	Contigs	Total bp	L50	N50	N50 ratio^b^	CDS	Plasmid (bp)		
PSU-1	USA, red wine	Sanger	1	1,780,517	1,780,517	1	0	1,878		CP000411	Mills et al. 2005
ATCC_BAA-1163	France, red wine	Sanger	61	1,748,994	61,665	10	311	1,835	pLo13 (3,948)	AAUV00000000	NCBI
AWRIB129	France	Illumina	42	1,729,193	135,603	5	311	1,780		AJTP00000000	Borneman et al. 2012a
AWRIB202	Australia	Illumina	36	1,840,757	137,205	4	288	1,914		AJTO00000000	Borneman et al. 2012a
AWRIB304	Australia	Illumina	36	1,852,239	137,195	4	288	1,928		AJIJ00000000	Borneman et al. 2012a
AWRIB318	Australia	Illumina	26	1,808,452	241,841	3	199	1,879		ALAD00000000	Borneman et al. 2012a
AWRIB418	USA	Illumina	34	1,838,155	177,870	4	255	1,887		ALAE00000000	Borneman et al. 2012a
AWRIB419	France	Illumina	46	1,793,208	135,466	5	377	1,861	pOENI-1 (18,431)	ALAF00000000	Borneman et al. 2012a
AWRIB422	France, Champagne	Illumina	32	1,814,530	228,430	3	309	1,893	pOENI-1v3 (21,317)	ALAG00000000	Borneman et al. 2012a
AWRIB429	Italy	Illumina	58	1,927,702	85,101	8	363	2,042	pOENI-1v2, (21,926)	ACSE00000000	Borneman et al. 2012a
AWRIB548	France, champagne	Illumina	29	1,835,383	228,488	3	251	1,929		ALAH00000000	Borneman et al. 2012a
AWRIB553	France	Illumina	32	1,759,113	229,549	3	309	1,814		ALAI00000000	Borneman et al. 2012a
AWRIB568	Australia	Illumina	31	1,874,865	137,199	4	209	1,968	pOENI-1v2 (22,031)	ALAJ00000000	Borneman et al. 2012a
AWRIB576	Australia	Illumina	28	1,877,204	241,903	3	233	1,964	pOENI-1v2 (22,005)	ALAK00000000	Borneman et al. 2012a
IOEB_0205	France, champagne	454	42	1,795,037	157,775	4	399	1,879		AZHH00000000	This study
IOEB_0501	France, red wine	454	38	1,826,356	162,140	5	251	1,892		AZIP00000000	This study
IOEB_0502	France, red wine	Illumina	39	1,822,270	140,250	5	265	1,883		AZKL00000000	This study
IOEB_0607	France, red wine	454	122	1,815,356	140,050	5	2855	1,873	pOENI-1v2	AZKK00000000	This study
IOEB_0608	France, red wine	454	41	1,812,611	108,677	6	239	1,882		AZKJ00000000	This study
IOEB_1491	France, red wine	Illumina	42	1,772,571	96,930	7	210	1,852		AZLG00000000	This study
IOEB_8417	France	454	65	1,842,137	95,439	7	539	1,907		AZKH00000000	This study
IOEB_9304	France, cider	454	137	1,827,658	79,430	9	1,948	1,901		AZKI00000000	This study
IOEB_9517	France	454	56	1,743,782	86,291	8	336	1,824		AZKG00000000	This study
IOEB_9803	France	454	36	1,833,906	146,580	5	223	1,889		AZKF00000000	This study
IOEB_9805	France	454	57	1,843,445	138,815	6	485	1,912		AZKE00000000	This study
IOEB_B10	NA	Illumina	42	1,779,079	108,811	5	311	1,841		AZJW00000000	This study
IOEB_B16	France, champagne	454	45	1,793,397	108,273	6	293	1,875		AZKC00000000	This study
IOEB_C23	France, cider	Illumina	47	1,837,655	93,272	8	229	1,941		AZJU00000000	This study
IOEB_C28	France, cider	Illumina	130	1,804,864	92,742	8	1,983	1,905		AZLE00000000	This study
IOEB_C52	France, cider	Illumina	48	1,903,774	101,748	6	336	1,946		AZLF00000000	This study
IOEB_CiNe	NA	Illumina	60	1,790,871	63,847	9	340	1,863		AZJV00000000	This study
IOEB_L18_3	Lebanon, red wine	Illumina	44	1,735,746	90,241	6	279	1,790		AZLO00000000	This study
IOEB_L26_1	Lebanon, red wine	Illumina	26	1,794,099	154,085	4	143	1,860		AZLP00000000	This study
IOEB_L40_4	Lebanon, red wine	Illumina	61	1,731,377	121,479	4	869	1,800		AZLQ00000000	This study
IOEB_L65_2	Lebanon, red wine	Illumina	39	1,776,569	105,259	5	265	1,850		AZLR00000000	This study
IOEB_S277	France	454	69	1,741,397	63,100	9	460	1,798		AZKD00000000	This study
IOEB_S436a	NA	Illumina	44	1,764,184	107,495	5	343	1,829		AZLS00000000	This study
IOEB_S450	France	Illumina	37	1,762,120	149,059	5	237	1,826		AZLT00000000	This study
IOEB_VF	France	Illumina	48	1,782,542	107,495	5	413	1,854	pOENI-1 (18,332)	AZLM00000000	This study
S11	France, white wine	Illumina	40	1,833,247	102,852	6	227	1,898	pOENI-1v2 (21,926)	AZJX00000000	This study
S12	France, white wine	Illumina	35	1,813,617	136,768	6	169	1,856		AZLH00000000	This study
S13	France, red wine	454	66	1,814,452	67,856	8	479	1,870		AZKB00000000	This study
S14	France, red wine	Illumina	40	1,731,907	85,103	5	280	1,800		AZLI00000000	This study
S15	France, red wine	Illumina	37	1,740,731	101,942	5	237	1,784		AZLJ00000000	This study
S19	France, red wine	Illumina	65	1,810,386	97,002	7	539	1,889		AZLK00000000	This study
S22	France, white wine	454	43	1,810,137	141,242	5	327	1,883		AZKA00000000	This study
S23	England, white wine	Illumina	50	1,805,457	84,503	7	307	1,859		AZLL00000000	This study
S25	France, red wine	454	32	1,741,301	140,671	5	173	1,808		AZJZ00000000	This study
S28	France, red wine	454	46	1,843,403	90,157	7	256	1,924		AZJY00000000	This study
S161	Red wine	Illumina	35	1,789,533	108,729	5	210	1,850		AZLN00000000	This study
DSM_17330^c^	Japan, shochu residue	Illumina	1	1,833,925	1,833,825	1	0	1,841	Unnamed (8,313)	ATZG00000000	Borneman et al. 2012b
NRIC_0647^c^	Japan, shochu residue	Illumina	27	1,839,043	261,715	3	216	1,849	Unnamed (8,365)	JSAG00000000	This study
NRIC_0649^c^	Japan, shochu residue	Illumina	16	1,825,564	285,276	3	69	1,832	Unnamed (8,280)^d^	JSAH00000000	This study
NRIC_0650^c^	Japan, shochu residue	Illumina	16	1,785,288	282,363	3	69	1,790	Unnamed (8,365)	JSAI00000000	This study

Note.—NA, not available.

^a^IOEB, Faculty of Enology of Bordeaux; S, SARCO (Bordeaux, France); ATCC, American Type Culture Collection, DSM, Deutche Sammlung von Mikroorganismen und Zellkulturen Gmb (Germany); NRIC NODAI Research Institute Culture collection (Tokyo, Japan).

^b^N50 ratio = ((Contigs − N50)/N50) × Contigs.

^c^ *Oenococcus kitaharae* strain.

^d^Broken in two contigs.

### Genome Sequencing, Assembly, and Annotation

Thirty-six *O. oeni* and three *O. kitaharae* genomes were sequenced and assembled either by using Illumina sequencing technology and SOAPdenovo assembler (Macrogen, Seoul, Korea) or 454 sequencing technology and Newbler assembler (GeT-PlaGe Genotoul, Castanet Tolosan, France). Contigs shorter than 200 bp were discarded and final genomes were deposed on NCBI under the accession numbers listed in table 1. All genomes were annotated by RAST (Aziz et al. 2008), curated manually and possible pseudogenes were indicated. Curated genes were resubmitted to KAAS annotation server (Moriya et al. 2007) of the KEGG project to get an extra reference. Coding sequences (CDS) annotated by RAST and KAAS were classified according to their ortholog groups using OrthoMCL (Li 2003).

### Modeling of the Progression of the Pangenome

The composition of the core, eco and pangenomes were calculated according to the ortholog groups derived from orthoMCL. From i = 2 to 49 genomes, the composition was calculated by randomly picking i genomes and calculating the composition of the pangenome, iterating the process 49 times, with the restriction that the same combination of genomes cannot be chosen twice. For the 50 genomes altogether, the composition can be calculated only once.

### Detection, Analysis, and Distribution of Single Nucleotide Polymorphisms

Raw reads were mapped against the reference genome of strain PSU-1 with the program BWA bwasw (Li and Durbin 2010). Single nucleotide polymorphism (SNP) were extracted with SAMtools and BCFtools (Li et al. 2009). An independent mapping and extraction of the SNP was carried out with MUMmer nucmer (Kurtz et al. 2004), both for the already assembled public genomes and for the final assemblies of the genomes of this study. The 47,621 resulting SNP positions were parsed into a matrix containing the allele carried by each strain. The distribution of SNP among different groups of strains was determined by measuring the Shannon Entropy for each SNP with the formula H = −∑p(xi) log2p(xi), where p(xi) represents the probability of finding the allele xi in an arbitrarily defined group of strains. The entropy was calculated for the groups of strains “A,”” B,” “strain IOEB_C52,” “champagne,” and “cider” as defined in figure 2. A SNP was considered to be unique to a certain group of strains whenever its entropy (H) was equal to 0 for the given group. The effect of each SNP was analyzed by snpEff (Cingolani et al. 2012), using the public genome of PSU-1 as reference. SNP affecting noncoding zones were discarded for the snpEff analysis.

### Distribution of Orthologs

All the CDS from all the strains were assigned to ortholog groups according to orthoMCL v2.0.9. The output was parsed to a matrix containing the number of CDS assigned to each ortholog group for each strain. The distribution of CDS among the groups of strains was determined by measuring the Shannon Entropy of each ortholog group from a matrix, exactly in the same way as for SNPs, except that rows represent each group of orthologs, and every cell contains the number of CDS assigned to each ortholog group, as if it were an allele. The distance between genomes was measured by Canberra method from the same matrix used to calculate the entropy. Pheatmap R package (R Core Team 2013) was used to calculate the distance and visualize the results.

### Phylogenetic Reconstructions

MLST data were collected from each genome sequence by retrieving the sequences of seven house-keeping genes already reported (Bilhère et al. 2009) using BLAST (Altschul et al. 1997). A 3,463-bp concatenated sequence was produced for each strain and used to reconstruct a tree by the neighbor joining method with 1,000 bootstrap replications and the Kimura 2-parameter model with MEGA v5.2.2 (Tamura et al. 2011).

Artificial sequences of 47,621 bp were produced for each genome by concatenating all the SNPs from the SNP matrix (see above) and used to reconstruct a tree using exactly the same method and parameters as for MLST. The program Structure (Hubisz et al. 2009) was used to analyze the population structure, using the same SNP data. To choose an optimal *k* value, the program was run with *k* values ranging from 1 to 8, burning period of 10.000, 2.000 Markov chain Monte Carlo repetitions, and each step was iterated ten times. The *k* value that best fitted the model was selected for the definitive analysis.

Distances between genomes were calculated by ANIm, ANIb, and Tetra algorithms with JSpecies v1.1 (Richter and Rosselló-Mora 2009). The difference between ANIm and ANIb is that the latter works by cutting the genomes in 1,020 bp pieces and averages the best matches of an all-versus-all BLAST, whereas the former does not cut the genomes and searches the matches by MUMmer. The resulting similarity matrices were transformed into distance matrices and used to reconstruct trees by the neighbor joining method with MEGA v5.2.2.

All trees were further processed and plotted with APE R Package (Paradis et al. 2004).

## Results and Discussion

### General Features of 36 Newly Reported O. oeni Genomes

The general characteristics of the 36 genomes described in this study are listed in table 1, along with those of the 14 previously described genomes and 3 new sequences of the sister species *O. kitaharae.* The 36 strains associated with the genomes of this study were isolated from different products and regions and at different years. They were selected for the diversity of their origins and their phylogenetic position according to previous studies (Bilhère et al. 2009; Bridier et al. 2010; Favier et al. 2012). Among the total of 50 studied strains, most come from France (33), while some others come from Australia (5), Lebanon (4), United States (2), Italy (1), and England (1). Twelve are commercial starters that were initially isolated from wines but afterwards produced industrially. The 36 new genomes are representative of different products: red wine (18), white wine (4), champagne (2), and cider (4). Illumina and 454 technologies were used to produce 21 and 15 genomes, respectively. The assembled genomes are made of 26–137 contigs. The N50 ratio values of the genomes suggest that the quality of assemblies tends to be better for genomes sequenced by Illumina, which is consistent with previous studies (Luo et al. 2012). The range of the sizes of the 36 new assembled genomes (from 1,731,377 to 1,903,774 bp) falls in the range of the 14 previously reported genomes (from 1,729,193 to 1,927,702 bp). In the same way, the number of identified CDS in the new genomes falls in the same range, from 1,784 to 1,946, compared with the range from 1,780 to 2,042 for the previously reported genomes. We did not detect any pLo13-type plasmid in any of the new genomes, nor another cryptic plasmid, such as the one described for the strain ATCC_BAA-1163. However, three strains carry plasmids of the pOENI-1 family (Favier et al. 2012). The strain IOEB_C52 contains a contig with genes that are typical of conjugative plasmids: a complete set of the Trs proteins, conjugation proteins, integrases, and transcriptional regulators. Nevertheless, we found no evidence that this contig might be part of a plasmid rather than integrated in the chromosome. The tree *O. kitaharae* genomes produced here share very similar properties to that of the previously sequenced strain DSM_17330 (Borneman et al. 2012b) and contain the same plasmid.

### Pangenome of O. oeni

**Fig. 1.—:**
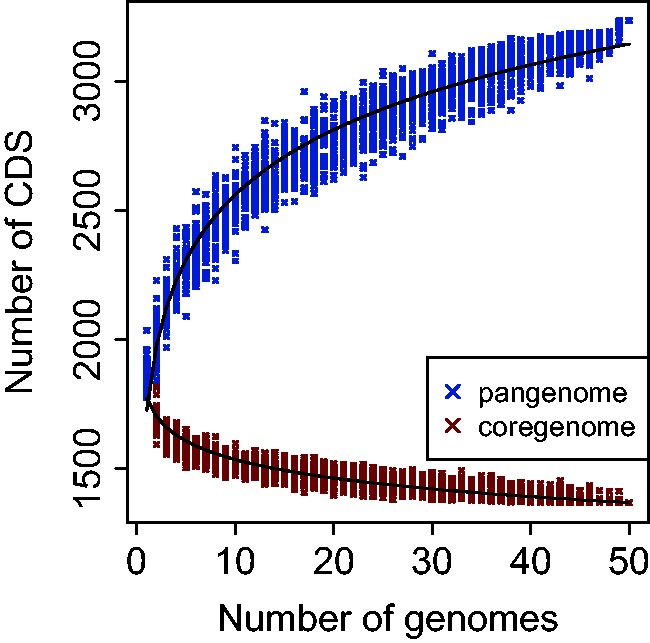
Progression of the core and pangenome of *O. oeni.* The progression on the composition of the core (red) and pangenome (blue) of *O. oeni* was computed by adding genomes one by one and iterating the process until reaching the 50 genomes.

To evaluate whether the pangenome (sum of all the genes of all the collected strains) (Medini et al. 2005; Tettelin et al. 2008) of the species has been fully represented, we determined the ortholog groups, analyzed the composition of the pangenome, and plotted the evolution of the coregenome (set of genes shared by all the strains) versus the pangenome from 1 to 50 strains (fig. 1). Tendency of the curves suggests that neither the coregenome nor the pangenome of the species has been fully represented yet. The pangenome for the 50 strains is represented by 3,235 CDS, distributed in 2,469 ortholog groups (table 2). The core genome is represented by 1,368 CDS, distributed in 1,160 orthologs. There are also 1,452 CDS that form the shellgenome (genes shared by only some strains) distributed in 902 ortholog groups, whereas 415 CDS belong to the cloud genome (genes present in only one strain). The size of the pangenome is consistent with previous studies that showed a pangenome size of 2,846 CDS for a group of 14 strains (Borneman et al. 2012a). However, the size of the coregenome is bigger than that of the fore mentioned study (1,165 CDS for the group of 14 strains), a divergence that is due to the different methods used to determine orthologs. Due to this divergence of the methods, if we recalculate the pan and coregenomes for the group of 14 strains we get a set of 2,639 and 1,512 genes, respectively.

**Table 2:** Pan and Coregenome of *O. oeni*

Total (50 strains)	Ortholog Groups	Total Genes
Coregenome	1,160	1,368
Shellgenome	902	1,452
Cloudgenome	407	415
Pangenome	2,469	3,235
Group A (37 strains)		
Coregenome	1,278	1,513
Shellgenome	653	1,047
Cloudgenome	190	191
Pangenome	2,121	2,751
Group B (12 strains)		
Coregenome	1,233	1,480
Shellgenome	504	807
Cloudgenome	282	293
Pangenome	2,019	2,580

### Population Structure of O. oeni

**Fig. 2.—:**
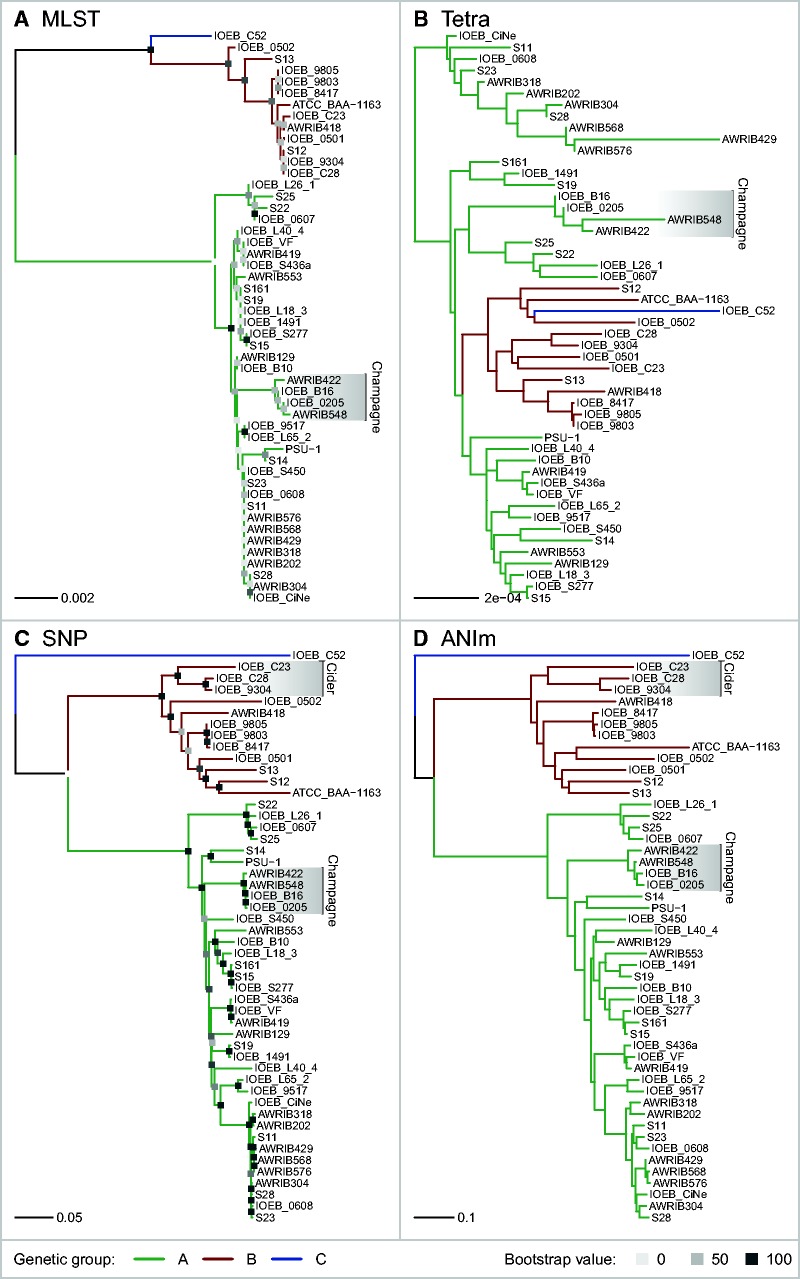
Phylogenetic and phylogenomic reconstructions of *O. oeni* by four different methods. Phylogenetic reconstruction by MLST was compared against phylogenomic reconstructions by Tetra, SNP, and ANIm. When possible, bootstrap values were calculated by doing 1,000 iterations (values indicated in bottom legend). Major genetic groups are indicated as in the legend. Strains coming from the same product (champagne, cider) are indicated when they form a single cluster.

The population structure of *O. oeni* was investigated by four methods based on different genomic properties: MLST, signature of tetranucleotides, SNP, and whole-genome alignment. A first phylogenetic tree, based on MLST data, was produced in order to compare with MLST trees reported previously (Bilhère et al. 2009; Bridier et al. 2010). The sequences of seven housekeeping genes were extracted from all of the 50 genomes and used to reconstruct a tree. In agreement with previous studies the MLST tree topology shows that the 50 *O. oeni* strains are distributed in two major genetic groups, A and B (fig. 2*A*). This tree, however, differs for strain IOEB_C52, which had been attributed to a third putative group C in the previous study (Bridier et al. 2010). Indeed, this strain is not clearly excluded from group B in the tree of figure 2*A*, although it branches apart from all other group B strains.

To evaluate the similarity of the genomes in terms of environmental pressure, we performed an analysis based on the genomic signature of tetranucleotides by Tetra algorithm (Karlin et al. 1997; Teeling et al. 2004; van Passel et al. 2006; Nishida et al. 2012). The genomic signature can change upon the action of selection pressure and environment and start diverging even between genomes with similar sequences (Pride 2003; Bohlin and Skjerve 2009; Bohlin et al. 2010), or inversely, environmental pressure can act as a driving force to keep the genomic signature stable even when different strains of a species can start to differ in their genomic sequence (Richter and Rosselló-Móra 2009). Therefore analyzing the 50 *O. oeni* genomes by Tetra was useful for confirming or refuting phylogenies based on other methods. The tree derived from the analysis shows strain IOEB_C52 as part of the group B, the latter being embedded inside the group A (fig 2*B*). It is likely that this phylogeny is incorrect because Tetra is less efficient to compare closely related genomes of a single species than distant genomes from different species. However, the fact that group B strains form a well-defined cluster in the tree constructed by Tetra throws stronger evidence in favor of the separation of the two groups A and B.

**Fig. 3.—:**
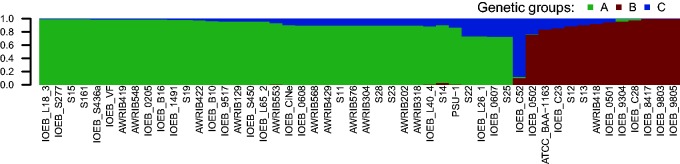
Population structure of *O. oeni.* Strains were probabilistically assigned to populations by calculating the frequencies of 47,621 SNP obtained from the SNP matrix (see Materials and Methods).

The SNP content of the genomes was analyzed to further investigate the population structure of *O. oeni.* Mapping all the genomes against the complete genome of strain PSU-1 revealed 47,621 SNP positions and a total of 48,230 alleles. A concatenated sequence of 47,621 bp was produced for each strain by extracting the alleles of all SNPs positions and the 50 sequences were used to reconstruct an unrooted tree by the neighbor joining method (fig. 2*C*). This tree has a slightly different topology from that of the MLST. Although they both agree in their two major branches A and B, the tree generated from SNPs clearly excludes strain IOEB_C52 from all rest, suggesting that this strain might actually be part of a third group C. Bootstrap values show a far more consistent tree than the one previously made by MLST. The fore mentioned trees are consistent with the results of previous studies (Bilhère et al. 2009; Borneman et al. 2012a), except for the newly sequenced strain IOEB_C52 that might be part of a genetic group that has not yet been described. SNP data was further processed by Structure software to infer the number of populations detected among the 50 strains. Structure is suited for inferring population structure since it works by probabilistically assigning individuals to populations by characterizing their allele frequencies at each locus. This method can be more reliable than distance-based methods such as neighbor-joining trees which do not let incorporate additional information, so they are more suited for exploratory analysis than for statistical inference (Pritchard et al. 2000). The result confirmed the presence of two populations corresponding to strains from groups A and B plus a third population represented by strain IOEB_C52 alone (fig. 3). For both A and B populations there is at least 70% of genetic contribution from their own group, and 0% to almost 25% contribution from group C. Strain IOEB_C52, the only individual of C group, has more than 80% of group C contribution and most of the contribution of the rest comes from B (fig. 3).

Finally, a phylogenetic tree based on whole-genome alignments was constructed using the average nucleotide identity (ANI) algorithm by MUMmer alignment (ANIm). This method calculates the distance between genomes by aligning the whole sequences using MUMmer and averaging the best matches. It can detect similarities that the SNP method would miss, especially when two strains being compared share a sequence that is absent in the reference strain used for SNP calling. Although the SNP and ANIm methods are strikingly different they produced trees sharing very similar topologies (fig. 2*C* and *D*). They both exclude strain IOEB_C52 from groups A and B. They also reveal a number of subgroups made of closely related strains. It is noteworthy that 4 strains isolated from Lebanon do not group together but are disseminated among diverse locations of branch A. In contrast, there are two clusters of strains isolated from the same type of product: three strains from cider and four strains from champagne. The latter were also grouped in the Tetra analysis, which confirms that they have started to evolve independently. Although three of these strains are industrial, IOEB_0205 is not, meaning that this genomic similarity might not be due to industrial selection. During the preparation of this manuscript the six new genomes of *O. oeni* strains isolated from “Nero di Troia” wine from cellars in the region of Apulia (Italy) were reported (Capozzi et al. 2014). A preliminary ANIm analysis showed that three of these strains are very close genetically and form a cluster in group A, whereas two other strains are dispersed in group A and the last strain falls in group B, with ATCC_BAA-1163 (data not shown)

### Evolution of Genetic Groups

**Fig. 4.—:**
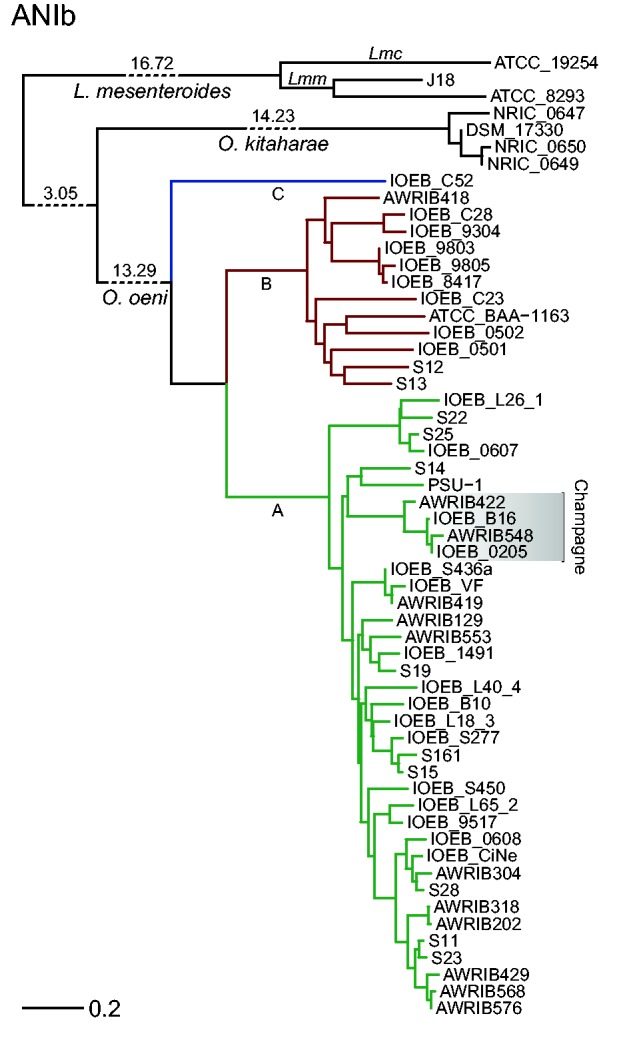
Phylogenomic reconstruction of *O. oeni* and its closest relatives by ANIb. The 50 *O. oeni* strains were branched to four strains of *O. kitaharae*, from which three were sequenced for this study, and three strains of *L. mesenteroides*, of which one corresponds to the *cremoris* subspecies (Lmc) and the other two correspond to *mesenteroides* (Lmm). The branches that separate the species were truncated for better display, which is represented by pointed lines. Numbers over the pointed lines indicate the total length of the respective branches. Distance is shown in terms of percentage of divergence according to ANI.

In order to evaluate the evolutionary relationships between *O. oeni* strains and between *O. oeni* and other species, an ANI tree was constructed using BLAST algorithm, known as ANIb (fig. 4). The tree was outgrouped by including three genomes of *Leuconostoc mesenteroides* subspecies *mesenteroides* and *cremoris*, and four genomes of the sister species *O. kitaharae* (table 1). Due to differences of sensibility between MUMmer and BLAST algorithms, discrepancies between trees constructed by both methods become more evident as genomes start to diverge (ANI < 90%). ANIm results are more robust when analyzing closely related genomes, but ANIb is preferable in this case since the compared genomes can have an ANI as low as 65%. A comparison of the previously published genome of *O. kitaharae* (Borneman et al. 2012b) and the three newly made genomes reported in this study reveals that they are rather homogenous at the sequence level in comparison to those of *O. oeni.* This is not surprising since all four strains were isolated from the same sample (Endo and Okada 2006), even if it is not uncommon to find genetically different strains in the same environment. The branch lengths of the reconstructed tree show that *O. oeni* strains are more divergent than strains of *L. mesenteroides* at the sequence level, although the latter are considered to form two subspecies (Hemme and Foucaud-Scheunemann 2004). However, sequence similarity alone is not enough to determine whether a set of strains corresponds to different (sub)species or not. In one hand, in order to be considered as a single species the genomes must share at least greater than 95% ANI (Thompson et al. 2013), which corresponds to the case of *O. oeni.* In the other hand, phenotypic characteristics can be at least partially predicted from genomic data in order to further classify the strains of a species (Amaral et al. 2014). This might be the case of the strains isolated from champagne and of IOEB_C52. The former shares a set of 27 unique SNP that generate truncate or longer proteins, or that skip the start codon. The affected genes are implied in diverse metabolic pathways which could at least partially explain this strains’ adaptation to champagne. They also have a cellulose 1,4-beta-cellobiosidase enzyme that does not match with the other strains according to the orthoMCL analysis. The strain IOEB_C52, at the sequence level, appears at the most basal position among *O. oeni* strains and has a set of 65 unique genes, some of them possibly explaining some of its technologic properties. However, because this is the only individual representing its putative group, the evidence to confirm that it might belong to a different class is weak. From the evolutionary point of view, this strain might represent a genetic group that preceded the advent of groups A and B, because domestication is also driven by a loss of genetic functions and a specialization. Interestingly this strain was isolated from cider as three other strains from group B. It is not surprising that *O. oeni* develops well in cider because cider is rather similar as wine regarding stress parameters: acidity, ethanol, polyphenols, and available substrates (sugars, malate, and citrate). The main difference is probably the total level of alcohol that rarely exceeds 6% in cider, whereas it is usually 11–14% in wine (Picinelli et al. 2000). Bacteria that naturally occur on fruits are exposed to low ethanol levels when overmaturated fruits are decomposed by the action of molds and yeasts. Therefore it is possible that the most ancient *O. oeni* strains, represented by strain IOEB_C52, were adapted to low ethanol containing environments, and that some strains of group B and most strains of group A have evolved to tolerate higher ethanol concentrations and to survive in wine. This likely represents a case of strain domestication because the wine environment exists only due to human activity. Domestication of *O. oeni* has been already reported (Douglas and Klaenhammer 2010); however, our results suggest that this domestication has not reached to the same level the strains of groups A, B, and C, which is reflected at the genomic level and confirmed by the population structure analysis. Because they group together, *O. oeni* strains from champagne have probably evolved a supplementary adaptive ability that could be the tolerance to the extreme acidity of this type of wine (pH ∼3.0). Domestication of other microorganisms in wine has also been observed for some species belonging to the *Saccharomyces sensu stricto* complex (Sicard and Legras 2011), such as *Saccharomyces cerevisiae* (Fay and Benavides 2005; Legras et al. 2007; Albertin et al. 2009) and *Saccharomyces uvarum* (Almeida et al. 2014).

### Occurrence of Group A and B Strains in Wine

To compare the occurrence of group A and B strains in wine, a PCR assay was developed to detect specifically group A or B strains with two couples of primers targeting specific genes of each group. A first screening was performed to detect group A and B strains in 65 wines collected during MLF. The PCR test showed positive results for group A strains on the 65 wines, but no detectable signal for group B strains (table 3). This indicates that large populations of group A strains were present in all these wines. However, it is possible that minor and undetectable populations of group B strains were also present. To test this possibility, a second PCR screening was performed on 110 *O. oeni* strains isolated from wines during MLF. None of the strains from this collection correspond to the genomes reported in this work. A total of 105 strains from group A and only 5 strains from group B were detected. This suggests that group A strains are the best adapted to wine conditions, and a result that is consistent with the presence of cider strains in group B and champagne strains in group A. However, it is not surprising to detect some group B strains in wine since they have been previously detected in Spanish wines (Bordas et al. 2013). It would be interesting to determine if group B strains are occasionally encountered in diverse environments or if they predominate in some regions or types of wines.

**Table 3:** Occurrence of *O. oeni* A and B in Wine during MLF by PCR Test

Genetic group	Total DNA	Colony PCR
A	65	105
B	0	5

### Core and Pangenomes of A and B Strains

To better understand the role of the genetic variability in the evolution of *O. oeni*, the species was analyzed in terms of the coregenome, shellgenome, and cloudgenome of groups A and B separately. The core and pangenomes of the 37 group-A strains and 12 group-B strains were determined by plotting curves as described above for the whole *O. oeni* population. The coregenome was bigger for group A than for group B (table 2). This was not expected, since the general tendency is that the bigger a group is, the smaller becomes the coregenome, only if the genetic diversity is equivalent between the groups being compared. It is difficult to discuss on the composition of the shell and cloudgenomes, since adding more strains to a group raises the probability of finding new genes, but it also raises the probability of a gene formerly considered as unique to be found in a new strain, becoming part of the shellgenome. Thus, the numbers in the shell and cloudgenome tend to be more stable than those of the pan and coregenome. Taking that into account, we can observe that the cloudgenome of group B is bigger than group A’s, suggesting a greater genetic diversity. When analyzing the pangenome, the situation was more consistent because the larger group A had the bigger pangenome. However, when the pangenome of group A is considered for 12 randomly selected strains to equal the size of group B, the pangenome contains only 2,450 ± 55 genes, which is smaller than the pangenome of group B, and the coregenome consists of 1,563 ± 14 genes, which is bigger than that of B. These results confirm that strains of group B are genetically more diverse than strains of group A. Group B strains might have had more time to diverge, whereas the strains of group A are more conserved, but at the same time more commonly found in wine. Also, the fact that the strains of group A have a narrower pangenome suggest that they might be in process of further domestication to wine-like environments. This is also supported by the fact that, despite being more numerous and commonly found in wine, group A strains are genetically closer between them than the group B strains, according to all the phylogenetic and genomic analyses previously mentioned. Both groups A and B lack the lanthionine biosynthesis proteins that are present in IOEB_C52 and other enzymes involved in the synthesis of some metabolites. Loss of genes with consequent auxotrophy, along with an augmented number of transporters, is another sign that the species has been domesticated (Douglas and Klaenhammer 2010).

### Specific Genetic Features of Groups of Strains

**Fig. 5.—:**
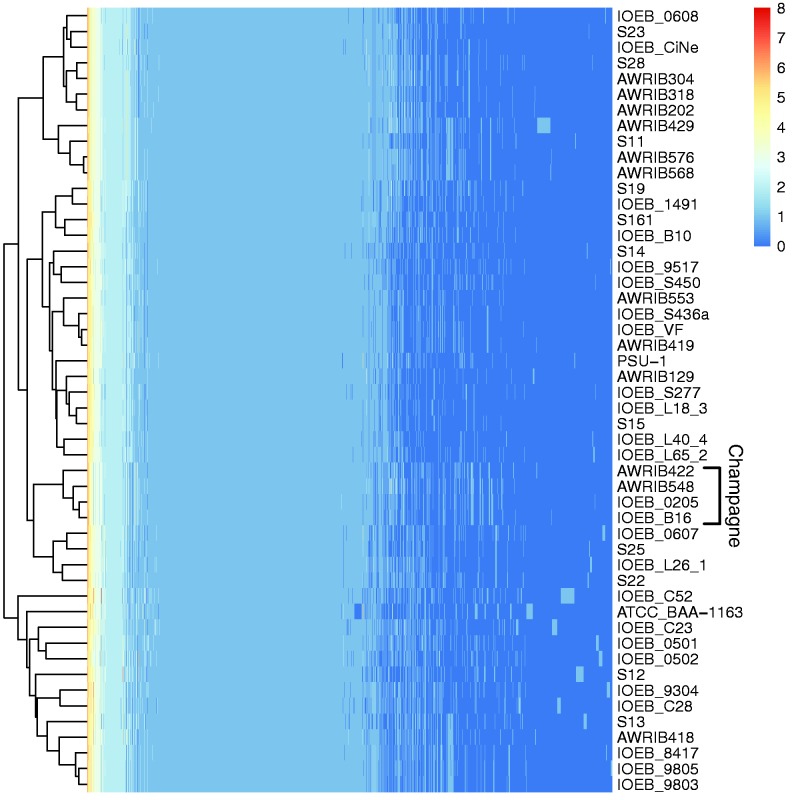
Cluster analysis on the ortholog groups of *O. oeni.* Ortholog groups are represented in the form of heatmap, where each cell displays the number of CDS contained in the group for each strain. The number of CDS of for each ortholog ranges from 0 to 8.

A search for specific genes and SNP was also performed in order to determine if some of them could explain some characteristics of the group where they are present. To determine whether the groups A and B differ by the absence or presence of specific genes, we performed a cluster analysis that depicts the distribution of the 2,469 ortholog groups of the *O. oeni* pangenome among the 50 strains (fig. 5). The resulting heat map reveals two major clusters for genetic groups A and B, with strain IOEB_C52 being the most external of cluster B. It is also possible to observe a clade made of strains that come from champagne. The genes specific of groups of strains were identified by calculating Shannon Entropy (H) for each ortholog group. A total of 94 orthologs specific to strains either of group A, B, champagne or strain IOEB_C52 were detected (table 4*A*). They encode hypothetical proteins, transcription regulators and proteins involved in diverse functions, but none that is obviously related to ethanol resistance (supplementary table S1, Supplementary Material online). Genes that are present exclusively in groups A or B are limited to hypothetical proteins. Genes unique to IOEB_C52 include, besides the Trs system mentioned before, a phosphoglycolate phosphatase, lanthionine biosynthesis proteins, transporters, sugar utilisation, and nucleotide metabolism proteins. At the same time, this strain lacks a set of five hypothetical proteins that are present in all the other strains. The four strains isolated from champagne share a unique set of nine genes, seven coding for hypothetical proteins, one for a primase–helicase, and one for cellulose 1,4-beta-cellobiosidase. They also lack, along with the strain IOEB_S450, a gene encoding an esterase C. The loss of this gene in two of the champagne strains had already been reported (Mohedano et al. 2014). A detailed list of all the discriminating orthologs among strains of group A, B, C, champagne and cider is shown in supplementary table S1, Supplementary Material online.

**Table 4:** Unique CDS and SNP of Groups of Strains of *O. oeni*

	By Genetic Group				By Product	
	A	B	C		Champagne	Cider
(*A*) Counts of Orthologs with H = 0						
No. of strains	37	12	1		4	3
Present orthologs	3	2	65		9	1
Absent orthologs	6	4	5		0	1
Total discriminating orthologs	9	6	70		9	2
(*B*) Counts of SNP with H = 0						
No. of strains	37	12	1		4	3
Noncoding zone	369	326	1,257		196	38
Synonymous	1,879	1,483	4,633		303	44
Nonsynonymous	0	446	1,625		559	49
Start lost	0	0	0		3	0
Stop lost	0	0	2		1	0
Stop gained	0	6	17		23	0
Total discriminating SNP	2,248	2,261	7,534		1,085	131

For the SNP analysis, a total of 48,230 alleles were extracted from 47,621 positions, giving a total of 13,144 specific SNP (with H = 0, table 4*B*). The strains of group A share 2,248 specific SNP, of which 1,879 affect coding zones. Because the SNP were mapped against the genome of the strain PSU-1 as reference, the molecular effect of all the SNP belonging to the same group of strains as PSU-1 are to be considered as synonymous. For the genetic group B, there is a total of 2,261 specific SNP, of which 1,936 affect coding zones. Among these, 446 are nonsynonymous and 6 are nonsense mutations, all of them truncating the proteins at less than one-third of their original length. The strain IOEB_C52, the only member of group C, has a total of 7,534 unique SNP, of which 6,287 affect coding zones, 1,625 are nonsynonymous, 2 are lost stop codons, and 17 are nonsense. There are also SNP that are characteristic of strains from certain products. For instance, the strains from champagne share a set of 1,085 SNP that are not found elsewhere and can be considered typical of this group. From these, 23 correspond to nonsense SNP, 3 to start lost, and 1 to a lost stop codon. Of the 23 nonsense mutations, 20 truncate the proteins at less than one-fourth of their original length, and the remaining three truncate them at less than one-third. Although some of these mutations affect hypothetical or viral proteins, many others affect genes that code for permeases, deiminases, decarboxylases, dehydrogenases, kinases, transferases, RNases, and other proteins which could eventually explain the adaptation of those strains to a different environment. Strains of champagne have a high number of unique SNP in comparison to other groups with the same number of strains. For instance, the three strains from cider in group B share only 131 unique SNP, with 93 affecting coding zones: 44 are synonymous mutations and 49 are nonsynonymous. A detailed list of all the SNP affecting start and stop codons on the fore mentioned groups is shown in supplementary table S2, Supplementary Material online.

## Conclusion

Revisiting the population structure of the *O. oeni* species by comparative genomics confirmed the distribution of strains reported in previous studies, that is, two major groups, namely A and B, and a number of subgroups. The predominance of group A strains in wine could argue in favor of the existence of subspecies, however group B strains are occasionally detected in wine and there is not a clear phenotypic divergence between strains from both groups, so that the definition of subspecies is still premature. A phylogenomic reconstruction including genomes of closely related species revealed one strain that is possibly member of an ancestral group at the origin of all other strains. This analysis, along with the distribution of orthologs, and the presence of unique genes and SNP, agree with the idea that *O. oeni* is a species that has been domesticated to cider and wine. Probably the group A has appeared as a new group with a fitness that lets it dominate wine-like environments better than group B and C. The narrowness of its pangenome in comparison to that of group B supports the idea that group A strains have been further domesticated than the others. The presence of unique genes and SNP could possibly explain some features of certain groups of strains (e.g., those coming from champagne).

## Supplementary Material

Supplementary tables S1 and S2 are available at *Genome Biology and Evolution* online (http://www.gbe.oxfordjournals.org/).
